# Analysing clinical reasoning characteristics using a combined methods approach

**DOI:** 10.1186/1472-6920-13-144

**Published:** 2013-10-29

**Authors:** Michele Groves, Marie-Louise Dick, Geoff McColl, Justin Bilszta

**Affiliations:** 1 Faculty of Health Sciences, The University of Queensland, Queensland, Australia; 2 School of Medicine, The University of Queensland, Queensland, Australia; 3 Melbourne Medical School, The University of Melbourne, Melbourne, Australia

**Keywords:** Clinical reasoning, Medical, Diagnosis, Assessment, Evaluation, Medical education, Clinical skills

## Abstract

**Background:**

Despite a major research focus on clinical reasoning over the last several decades, a method of evaluating the clinical reasoning process that is both objective and comprehensive is yet to be developed.

The aim of this study was to test whether a dual approach, using two measures of clinical reasoning, the Clinical Reasoning Problem (CRP) and the Script Concordance Test (SCT), provides a valid, reliable and targeted analysis of clinical reasoning characteristics to facilitate the development of diagnostic thinking in medical students.

**Methods:**

Three groups of participants, general practitioners, and third and fourth (final) year medical students completed 20 on-line clinical scenarios -10 in CRP and 10 in SCT format. Scores for each format were analysed for reliability, correlation between the two formats and differences between subject-groups.

**Results:**

Cronbach’s alpha coefficient ranged from 0.36 for SCT 1 to 0.61 for CRP 2, Statistically significant correlations were found between the mean f-score of the CRP 2 and total SCT 2 score (0.69); and between the mean f-score for all CRPs and all mean SCT scores (0.57 and 0.47 respectively). The pass/fail rates of the SCT and CRP f-score are in keeping with the findings from the correlation analysis (i.e. 31% of students (11/35) passed both, 26% failed both, and 43% (15/35) of students passed one but not the other test), and suggest that the two formats measure overlapping but not identical characteristics. One-way ANOVA showed consistent differences in scores between levels of expertise with these differences being significant or approaching significance for the CRPs.

**Conclusion:**

SCTs and CRPs are overlapping and complementary measures of clinical reasoning. Whilst SCTs are more efficient to administer, the use of both measures provides a more comprehensive appraisal of clinical skills than either single measure alone, and as such could potentially facilitate the customised teaching of clinical reasoning for individuals. The modest reliability of SCTs and CRPs in this study suggests the need for an increased number of items for testing. Further work is needed to determine the suitability of a combined approach for assessment purposes.

## Background

Clinical reasoning has been the focus of research for much of the past thirty years. This has been due as much to an inherent fascination with the topic itself as to the need to reduce the high incidence of adverse events due to missed and delayed diagnoses [1,2]. Indeed, the patient safety literature abounds with studies which describe common types of diagnostic error [3,4] but few, if any, propose a way of identifying these errors in practice and approaches to remediation [5]. Nevertheless, there is now, not only a much enhanced understanding of the cognitive processes involved in diagnosis and their relationship to knowledge [6], but also an increased focus on developing and enhancing clinical reasoning skills in students and practitioners [7].

In pursuit of this goal, numerous strategies have been devised to teach and to learn the diagnostic process and develop clinical reasoning skills, using both cognitive and formulaic approaches (such as heuristics and decision trees) [8-13]. Commonly (and perhaps understandably), the indicator of success in these teaching approaches is diagnostic accuracy with relatively little emphasis being placed on the need to develop a sound underpinning reasoning process. To date, a valid, reliable and objective method of identifying and evaluating an individual’s clinical reasoning characteristics and ability remains elusive [14].

In the absence of such a gold standard, developing a suite of methods able to evaluate one or more aspects of the clinical reasoning process may bring the achievement of this goal closer. Two possible, already established, methods are the Clinical Reasoning Problems (CRPs) [15] and the Script Concordance Test (SCT) [16]. These methods have some attributes in common, but generally are complementary with regard to their theoretical framework and assessment approach. Both these methods have been used in a variety of contexts and have demonstrated reliability and validity as tests of clinical reasoning skill in medical students and practitioners [15,16].

The CRPs aim to assess skill in diagnostic hypothesis generation as well as clinical data identification and interpretation, and thus provide a detailed and comprehensive evaluation of the clinical reasoning process. Each CRP describes a patient’s presentation, history and physical examination findings and respondents are asked to nominate the two most likely diagnoses based only on the information provided. For each nominated diagnosis, participants are asked to choose, from a provided list of clinical features, those features they considered important in reaching their diagnosis, as well as a weighting (positive or negative) for each which best describes its influence on their decision. A CRP score consists of three scales based on the marks for the diagnoses (d-mark), for feature identification and interpretation (f-mark) and total mark (d-mark + f-mark). When administered using the web-based version, respondents are also given immediate qualitative feedback in the form of access to the responses of the expert reference group which forms the basis of the marking scheme. CRPs therefore are able to be used for both teaching and assessment purposes [17].

The SCT has been well-described in several studies [16,18,19]. In contrast to the CRPs, SCTs focus specifically on clinical data interpretation. Their design allows weaknesses in this aspect of the reasoning process to be identified. As with the CRPs, SCTs use a case-based format and consist of a clinical scenario followed by up to five questions of the “if this…..then that…” type. Each question provides a possible diagnosis based on the scenario, followed by additional clinical information. Respondents are asked to indicate the impact of this information on the likelihood of the suggested diagnosis being correct, using a five-point scale from -2 (very unlikely) to +2 (very likely).

The scoring schemes for both methods are derived from the responses of a reference group with the highest marks being awarded to those responses which are closest to the majority panel responses.

Both the CRPs and SCTs have been used with large cohorts of medical students [20,21]. However, although a comprehensive assessment of clinical reasoning, CRPs are relatively time-consuming as each case requires approximately 10 minutes to work through, and 15–20 cases are required for good reliability. On the other hand, SCTs are more time-efficient requiring only a few minutes per case and it has been shown that 30–80 questions in the SCT format are needed to provide good reliability [19]. However, the cases are more narrowly focussed than the cases used in CRPs, as a number of diagnostic hypotheses (usually five) are included in each scenario. Thus, in a given time period, SCTs are able to test clinical data interpretation over a wider range of possible diagnoses than CRPs.

We speculated that using SCTs as a screening method to initially identify students with weak clinical reasoning, followed by a more comprehensive evaluation of those students using the CRPs, might provide an efficient yet targeted appraisal of their clinical reasoning process. The resulting detailed clinical reasoning profile could then potentially be used to design customised remediation activities for individual students. For such a combined approach to work however, there would need to be evidence of a partial correlation between total SCT and CRP scores, and a stronger correlation between total SCT score and the f-subscale of the CRPs.

Consequently, the aim of this study was to test the compatibility of the SCTs and CRPs used in a combined approach and whether this approach would provide a valid, reliable and comprehensive analysis of clinical reasoning characteristics that could subsequently be used to facilitate the development of customised teaching of medical students.

## Methods

### Subjects

Three groups of subjects were recruited on a voluntary basis as required for ethical approval of the study by the participating institutions. The first subject-group consisted of general practitioners associated with two Australian medical schools. The other two subject groups were third and fourth (final) year students enrolled in each university’s medical program.

### Instruments

The CRPs used in this study were those developed and evaluated previously^15^. They consisted of 20 clinical diagnostic scenarios, divided into two sets of 10 cases, labelled CRP 1 (cases 1–10) and CRP 2 (cases 11–20). The content of both sets were similar in that they covered a range of patient demographics and contexts representative of the type of common, undifferentiated clinical presentations that final year medical students would expect to encounter after graduation. (e.g. each set contained one case relating to the cardiovascular system, one relating to the respiratory system, etc.).

Each CRP scenario was also re-formatted as an SCT comprising the case and five questions. This resulted in two corresponding sets of SCTs of 10 cases and 50 questions each, labelled SCT 1 (cases 1–10) and SCT 2 (cases 11–20). Thus, each set of 10 clinical diagnostic scenarios was available in both CRP and SCT format (labelled CRP 1, SCT 1, CRP 2, SCT 2 respectively). The marking schemes for both formats (CRPs and SCTs) were drawn from the responses of an expert reference group of 21 experienced Australian GPs / family doctors [15].

An example of one clinical scenario presented in both CRP and SCT formats is provided in Additional file 1.

### Procedure

The study used a cross-over design in which one set of 10 CRPs was matched with its complementary set of 10 SCTs, thus forming two test-groups: CRP1/SCT 2 and SCT1/CRP2. Participants were allocated alternately to one of these two test-groups, so that they completed all 20 cases, half in CRP format and half in SCT format. Participants were emailed their allocated set of SCTs, as well as a login to access their CRPs online at a dedicated website. They were then asked to complete and submit both sets of questions electronically within three weeks. Completion time was estimated at about 90 minutes for the CRPs and about 30 minutes for the SCTs, bringing the expected total testing time to approximately two hours. All responses were automatically scored on submission and both scores and feedback provided to the participant. This was immediate in the case of the CRPs through access to the collated responses of the expert reference panel. For the SCTs, participants were provided with a comparison of their responses with the expert panels’ “best” answers, by return email.

### Data analysis

Statistical analysis was undertaken using SPSS 20. The Shapiro-Wilk statistic was calculated to determine distribution and Levene’s test to determine the homogeneity of variances. Reliability was calculated using Cronbach’s alpha coefficient for internal consistency. Evidence of construct validity was assessed by calculating the correlation between total SCT scores and CRP feature (“f-score”) and total scores. Differences between subject- groups were analysed using one-way analysis of variance (ANOVA). Finally, if the SCTs are to have utility as a screening technique, it is necessary to ensure that SCT scores are able to predict subsequent performance in the CRPs. In the absence of a criterion-referenced pass mark and to approximate a 50% score, the second quartile of the total score for the SCTs and the f-score for the CRPs was chosen as the notional pass mark. Using this figure, the number and proportion of students passing and failing the SCTs and CRPs was calculated.

## Results

From a total of 17 GPs and 202 students who agreed to participate in the study, CRP and/or SCT responses were received from 12 GPs (71%) and 119 students (59%). In the CRP1/SCT2 stream, these consisted of eight GPs, 20 Year 4 and 44 Year 3 students; in the SCT1/CRP2 stream, there were four GPs, 22 Year 4 and 33 Year 3 students. Additionally, 57 sets of SCTs were incomplete and removed from further analysis. Thus, the final analysis was based on 131 sets of CRPs and 74 sets of SCTs across all subject-groups.

### Descriptive statistics

The mean scores, standard deviations and distribution for all sets of CRPs and SCTs are shown in Table 1. The results of the Shapiro-Wilk test for normality, calculated on the combined group scores indicated that all data, with the exception of CRP 1 scores were normally distributed, thus justifying the use of parametric statistical analyses. Calculation of Levene’s statistic indicated that variances from the mean were homogenous across tests, again with the exception of CRP 1.

**Table 1:** Descriptive statistics and distribution over all Cohorts

**Test**	**N**	**Maximum total score**	**Mean total score (SD)**	**% Mean total score**	**Shapiro-Wilk statistic (p)**	**Levene’s statistic (p)**
**CRP 1**	70	133	77.92 (11.64)	58.57	0.96 (0.02)	4.25 (0.02)
**CRP 2**	51	135	77.22 (9.76)	57.20	0.99 (0.87)	2.01 (0.14)
**SCT 1**	32	51	28.90 (3.36)	56.67	0.95 (0.17)	1.86 (0.87)
**SCT 2**	42	50	25.14 (4.03)	50.28	0.97 (0.32)	0.21 (0.81)

Consequently, one-way ANOVA was used to compare differences between subject-groups (see Table 2). Results indicate that inter-subject-group differences were significant or approached significance for the CRPs but not for the SCTs. Contrast tests between pairs of subject-groups consistently showed significant differences in CRP performance across all scales (d-mark, f-mark and total mark) between the GPs and one or both student groups.

**Table 2:** Comparison of Means by Cohort

**Cohort**	**CRP 1**				**CRP 2**				**SCT 1**		**SCT 2**	
	**N**	Mean total d-mark (SD)*	**Mean total f-mark (SD)**	**Mean total mark overall (SD)**	**N**	Mean total d-mark (SD)*	**Mean total f-mark (SD)**	**Mean total mark overall (SD)**	**N**	**Mean total mark (SD)**	**N**	**Mean total mark (SD)**
GPs	8	13.24^a^ (1.27)	71.61^a^ (6.57)	84.85^a^ (6.90)	2	15.02^a^ (1.52)	78.26^a^ (5.17)	93.26^a^ (6.68)	4	26.77 (2.27)	8	25.11 (3.98)
Year 4 students	19	12.69^a^ (1.25)	66.80^a^ (8.13)	79.50^a^ (8.90)	22	12.06^b^ (1.31)	65.67^b^ (5.78)	77.72^b^ (6.71)	12	28.96 (4.00)	13	24.41 (4.29)
Year 3 students	43	11.90^b^ = (1.93)	64.03^b^ (11.18)	75.93^b^ (12.89)	27	11.72^b^ (1.88)	63.91^b^ (9.33)	75.62^b^ (11.06)	16	29.39 (3.01)	21	25.60 (4.03)
One-Way ANOVA		F[3,69] = 2.90	F[3,69] = 2.10	F[3,69] = 2.31		F[3, 50] = 3.75	F[3, 50] = 3.13	F[3, 50] *=* 3.39		F[3, 31] =0.97		F[3, 41] = 0.34
		p = 0.06	p = 0.13	p = 0.11		p = 0.03	p = 0.05	p = 0.04		p = 0.39		p = 0.71

*Means within columns with a superscript a or b in common do not differ significantly (as shown by Contrast Tests).

### Reliability

Table 3 shows Cronbach’s alpha coefficient for internal consistency for each group of tests. Over all cohorts, Cronbach’s alpha was 0.61 for CRP 1, 0.56 for CRP 2, 0.36 for SCT 1 and 0.60 for SCT 2. As would be expected, reliability increased when calculated using all 20 cases - to 0.93 for the CRPs and to 0.63 for the SCTs. Deleting any single problem from the analysis did not produce a substantial change in reliability.

**Table 3:** Reliability analyses

	**No. of items**	**No. of responses**	**Cronbach’s α**
SCT 1	51	32	0.357
SCT 2	50	42	0.596
CRP 1	40	66	0.613
CRP 2	40	48	0.560

### Construct validity

The mean scores for all CRPs and SCT cases were calculated and the Pearson correlation coefficients determined. Correlation between total CRP and SCT scores ranged from 0.46 – 0.49, and from 0.44-0.69 between CRP f-score and total SCT score (see Table 4). Statistically significant correlations were found between mean CRP 2 f-score and SCT 2, mean total combined CRP score and mean combined SCT score, and between combined CRP mean f-score and combined SCT score.

**Table 4:** Correlation analyses between CRPs and SCTs

		**Pearson’s r-coefficient**	
	**No. of cases**	**Mean F-score vs mean SCT score**	**Mean total score vs mean SCT score**
CRP 1 & 2	20	0.570**	0.467*
CRP 1	10	0.439	0.486
CRP 2	10	0.685*	0.461

*p < 0.05; **p < 0.01.

Using the described notional pass mark, a total of 11 out of 35 (31%) of students passed both SCT and CRP (f-score) tests, and 9 (26%) students failed both tests. Of 16 students who failed the SCT, nine students (56%) failed the CRP f-score, but 7 students (44%) passed the CRP f-score; of 19 students who passed the SCT, 11 students (58%) passed the CRP f-score, whilst 8 (42%) failed the CRP f-score (Table 5).

**Table 5:** Pass-Fail Comparison based on second quartile SCT total score and second quartile CRP f-score

	**Pass CRP**	**Fail CRP**	
Pass SCT	11	8	
Fail SCT	7	9	
			Total N = 35

## Discussion

This study has explored the compatibility of two methods of evaluating clinical reasoning, the CRPs and SCTs, to profile the clinical reasoning characteristics of students and clinicians.

Overall, the results suggest that CRPs discriminate well between levels of expertise; this may be because reflecting *back* on the features considered in generating a diagnostic hypothesis is less difficult once a provisional decision has been made. Interestingly, the SCTs were less able to discriminate between levels of expertise; this finding is difficult to interpret as, for each question within a case, subjects are provided with both a possible diagnosis and related patient information, and are required only to interpret the specific clinical data provided. It is possible that part of the explanation lies in the voluntary nature of the student sample and the relatively low response rate (59%), possibly resulting in the more able students being disproportionately represented and leading to smaller differences in SCT scores between them and the experts than would normally be expected. Alternatively, the cases were designed to cover a range of patient demographics and presentations and it may be that content specificity at all levels of expertise was responsible for some of the difficulty in discriminating between subject-groups. A third possibility is that medical students and GP clinicians are more readily able to recall the medical knowledge needed to interpret clinical findings to a diagnosis, once the diagnosis has been specified.

The moderate correlation between SCTs and CRPs suggests that the two methods measure overlapping but not identical reasoning characteristics. As would be expected, a higher correlation was found between the CRP scale related to data interpretation (f-score) and SCT score as this is the aspect of greatest convergence. Despite this correlation however, just 20 out of 35 students (65%) consistently passed or failed both the SCT and CRP f-score tests, while student performance in the SCT test was not consistent with performance in the CRP f-score for 35% of students. Additionally, only 56% of students who failed the SCT test also failed the CRP f-score, indicating it was not a useful predictor of performance in CRP f-score. Small participant numbers are likely to have influenced these results, and it is possible that a larger sample size combined with a more systematic approach to setting the pass mark for both tests may improve the correlation between them. Cronbach’s alpha calculations show that CRPs have acceptable reliability, taking into account the semi-qualitative nature of the measure. While it is likely that reliability would improve if the number of problems per set were increased, this would mean extending the assessment period which may decrease feasibility and participation. The modest reliability of the SCTs found here is puzzling in that it is not consistent with previous studies which have calculated an average alpha coefficient of approximately 0.78 for 30–80 items [19]. Again, a possible explanation may be the relatively small number of cases used in the current study; further investigation is required to determine the influence of the number of cases on reliability.

The study’s findings are limited by the small number of subjects (particularly GPs), and the availability of complete sets of data for some of the analyses. While this is somewhat unavoidable due to the requirement for ethical approval that participation by both students and GPs be voluntary, it does mean larger trials are needed before the reliability and validity of this approach can be firmly established. In hindsight, it may also have been useful to include a self-report measure of clinical reasoning, such as the Diagnostic Thinking Inventory [22], to encourage self-reflection and analysis, thereby increasing individuals’ understanding of their own reasoning process in relation to that of diagnostic experts. Future work could explore the benefit of incorporating self-reporting measures to further emphasise the importance of metacognition in diagnostic expertise.

## Conclusion

Our findings suggest that using different but complementary methods of evaluating clinical reasoning provides a more detailed and qualitative appraisal than either the CRPs or SCTs alone. The SCTs are a practical, valid and time-efficient method of assessing interpretation of clinical data with respect to a given provisional diagnosis in large cohorts; whereas CRPs provide a more comprehensive picture by evaluating individual ability in diagnostic hypothesis generation and data synthesis, as well as data interpretation. While both tests assess data interpretation, this study demonstrates that results can vary depending on the way this is done. This, in combination with the low level of agreement in performance between the two methods suggests that they are likely to be most useful for teaching rather than assessment purposes. Important features of both techniques are that they provide immediate quantitative and/or qualitative feedback. Used together, they can provide a more comprehensive analysis of clinical reasoning ability that is necessary to develop customised remediation of specific identified weaknesses in three important aspects of the diagnostic process - hypothesis generation and clinical data synthesis and interpretation.

In summary, although the findings of this study suggest that using a two-stage approach provides a more comprehensive evaluation of clinical reasoning than either the SCT or CRPs alone, the choice of methods is critical particularly if it is to be used for assessment purposes.

## Competing interests

The authors declare that they have no competing interests.

## Authors’ contributions

MG designed the study, developed the methodology, coordinated recruitment of participants and data collection, analysed the data and wrote the draft paper. MLD assisted with the study’s design and conduct, recruited the doctors and students at the University of Queensland and contributed to the data analysis and writing of the paper. GMcC assisted in the study design and recruitment of students at the University of Melbourne and contributed to the data analysis and writing of the paper. JB coordinated student recruitment and data collection at the University of Melbourne and contributed to the writing of the paper. All authors read and approved the final manuscript.

## Pre-publication history

The pre-publication history for this paper can be accessed here:

http://www.biomedcentral.com/1472-6920/13/144/prepub

## Supplementary Material

Additional file 1 Example of CRP and SCT Formats. Click here for file
