# Supplementary material for: Analysing clinical reasoning characteristics using a combined methods approach
Source: BMC Med Educ. 2013 Oct 29;13:144. doi: 10.1186/1472-6920-13-144 (PMC4231458; doi:10.1186/1472-6920-13-144)
Supplement: Additional file 1 — Example of CRP and SCT Formats. [file 1472-6920-13-144-S1.pdf]

CRP Format

Russell M is a 55 year old architect who presents for a check-up. He has noticed that he becomes breathless easily, even after mild exercise. He also mentions that, while he has had a bit of a morning cough for the last few years, it seems to have been more severe and frequent in the last 3 or 4 months. He is also finding that he has to go to the toilet several times during the night.

Russell is a regular although infrequent patient of your practice. He does not smoke, and drinks moderately. He has always been a bit overweight, but has lost weight since you last saw him. He has a history of high blood pressure for which he takes captopril. During a period of unemployment 10 years ago, he developed insomnia which still bothers him occasionally. Other medical history includes successful repair of an inguinal hernia when he was 18 and a bout of whooping cough 2 years ago.

On examination, Russell's BP is 150/90; his respiratory rate is 20/min with widespread expiratory wheezing, his heart rate is 90 bpm with a mildly displaced apex beat. You note palmar erythema

---

1. Based on the above information, what do you think is the most likely diagnosis in this patient?
2. Using the list of clinical features and the key below, please select those (and only those) that you considered in reaching your diagnosis and the weighting option which best describes its influence on your decision.

   Clinical Features

   Shortness of breath
   Worsening morning cough
   Nocturia
   Non-smoker
   Alcohol intake
   Weight loss
   Blood pressure
   Captopril
   Insomnia
   Hx whooping cough
   Respiratory Rate
   Expiratory Wheeze
   Heart rate
   Displaced apex beat
   Palmar erythema

3. *If this diagnosis proved incorrect, what would your next choice be?*
4. *Again, please select only those clinical features that you considered in reaching your diagnosis and the weighting option which best describes its influence on your decision.*

   Clinical Features

   Shortness of breath
   Worsening morning cough
   Nocturia
   Non-smoker
   Alcohol intake
   Weight loss
   Blood pressure
   Captopril
   Insomnia
   Hx whooping cough
   Respiratory Rate
   Expiratory Wheeze
   Heart rate
   Displaced apex beat
   Palmar erythema

Key

+3 = strongly supportive; +2 = moderately supportive; +1 = slightly supportive; 0 = irrelevant;
-3 = strongly opposing; -2 = moderately opposing; -1 = slightly opposing.

SCT Format.

Clinical case: Russell M is a 55 year old architect who presents for a check-up. He has noticed that he becomes breathless easily, even after mild exercise. He also mentions that, while he has had a bit of a morning cough for the last few years, it seems to have been more severe and frequent in the last 3 or 4 months. He is also finding that he has to go to the toilet several times during the night.

The following hypotheses are considered:

	If you were thinking of	And then you find	This hypothesis becomes
**1-1**	**Left heart failure**	Respiratory rate is 20/min with widespread expiratory wheezing.	**-2 -1 [0] +1 +2**
**1-3**	**Left heart failure**	He has a history of well controlled high blood pressure for which he takes captopril.	**-2 [-1] 0 +1 +2**
**1-4**	**Asthma**	On physical examination, his heart rate is 90 bpm with a mildly displaced apex beat	**[-2] -1 0 +1 +2**
**1-5**	**Left heart failure**	palmar erythema	**-2 -1 [0] +1 +2**
**1-6**	**Asthma**	His respiratory rate is 20/min with widespread expiratory wheezing.	**-2 -1 0 [+1] +2**

Key

-2 = very unlikely; -1 = unlikely; 0 = neither less nor more likely; +1 = likely; +2 = very likely
